# Applying Digital Information Delivery to Convert Habits of Antibiotic Use in Primary Care in Germany: Mixed-Methods Study

**DOI:** 10.2196/18200

**Published:** 2020-10-07

**Authors:** Regina Poss-Doering, Lukas Kuehn, Martina Kamradt, Katharina Glassen, Michel Wensing

**Affiliations:** 1 Department of General Practice and Health Services Research University Hospital Heidelberg Heidelberg Germany

**Keywords:** antimicrobial resistance, educative digital solutions, health literacy, diffusion of innovations

## Abstract

**Background:**

Antimicrobial resistance is an important global health issue. In Germany, the national agenda supports various interventions to convert habits of antibiotic use. In the CHANGE-3 (Converting Habits of Antibiotic Use for Respiratory Tract Infections in German Primary Care) study, digital tools were applied for information delivery: tablet computers in primary care practices, e-learning platforms for medical professionals, and a public website to promote awareness and health literacy among primary care physicians, their teams, and their patients.

**Objective:**

This study is embedded in the process evaluation of the CHANGE-3 study. The aim of this study was to evaluate the acceptance and uptake of digital devices for the delivery of health-related information to enhance awareness and change habits of antibiotic use in primary care in Germany.

**Methods:**

This study used a convergent-parallel mixed-methods design. Audio-recorded semistructured telephone interviews were conducted with physicians, nonphysician health professionals, and patients in the CHANGE-3 program. Pseudonymized verbatim transcripts were coded using thematic analysis. In-depth analysis was performed based on the inductive category of information provision via digital information tools. Identified themes were related to the main postulates of Diffusion of Innovations theory (DIT) to provide an explanatory frame. In addition, data generated through a structured survey with physicians and nonphysician health professionals in the program were analyzed descriptively and integrated with the qualitative data to explore the complementarity of the findings.

**Results:**

Findings regarding the acceptance and uptake of digital devices were related to three postulates of DIT: innovation characteristics, communication channels, and unanticipated consequences. Participants considered the provided digital educative solutions to be supportive for promoting health literacy regarding conversion of habits of antibiotic use. However, health care professionals found it challenging to integrate these solutions into existing routines in primary care and to align them with their professional values. Low technology affinity was a major barrier to the use of digital information in primary care. Patients welcomed the general idea of introducing health-related information in digital formats; however, they expressed concerns about device-related hygiene and the appropriateness of the digital tools for older patients.

**Conclusions:**

Patients and medical professionals in German primary care are reluctant to use digital devices for information and education. Using a Diffusion of Innovations approach can support assessment of existing barriers and provide information about setting-specific preconditions that are necessary for future tailoring of implementation strategies.

**Trial Registration:**

International Standard Randomized Controlled Trial Number (ISRCTN) 15061174; http://www.isrctn.com/ISRCTN15061174.

## Introduction

### Background

Inappropriate use and prescribing of antibiotics are among the key contributing factors to antimicrobial resistance, which remains an important global health issue. Even after decades of scientific research and a range of improvement programs on the rational use of antibiotics, substantial room for improvement remains in promoting awareness and inducing necessary changes. In recent years, health services research worldwide has focused on interventions aimed at promoting the rational use of antibiotics, usually by providing information and education to health care workers, patients, and the public [1]. Thus, an evidence base has been established regarding the effects of educational interventions targeted at physicians that use internet-based training [2], reflection on habits and behaviors and peer exchange of information [3], changes in provider-patient communication toward participatory decision making [4,5], and involvement of the complete practice team to optimize organizational processes and to ease stress and strain on individuals [6]. Previous studies showed significant effects on decreasing rehospitalization rates and increasing patient empowerment by providing health literacy via tablet computers [7-9]. A recent review concluded that educating patients through smartphone or tablet apps improves treatment adherence and clinical outcomes and has positive effects on health care economics [10]. These studies were conducted in inpatient care settings and primarily aimed to deliver information to patients with postoperative conditions; meanwhile, factors that influence the acceptance and uptake of digital information delivery for promoting awareness and health literacy regarding the rational use of antibiotics in primary care remain less well researched.

**Figure 1:**
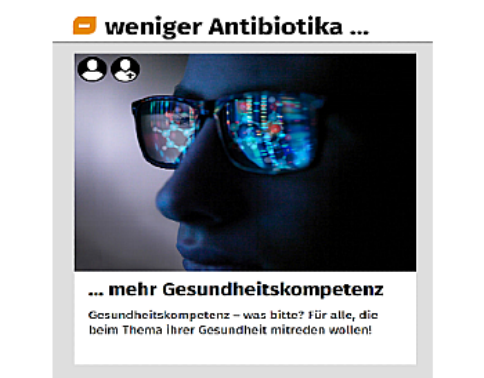
Screenshot of the study-specific website in German (“Fewer antibiotics…more health literacy”).

**Figure 2:**
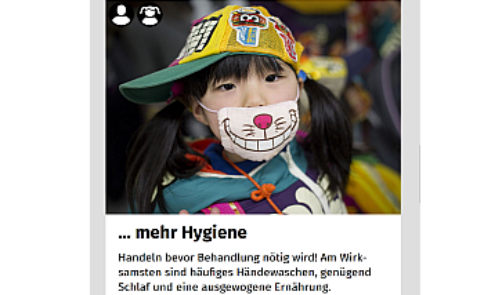
Screenshot of the study-specific website in German (“Fewer antibiotics…more hygiene”).

In Germany, the national agenda has reinforced policies to restrain the prescription of antibiotics [11]. As in many other countries, approximately 90% of antibiotics are prescribed in ambulatory care, mainly by general practitioners (GPs) [12] and most commonly during 41% of consultations for acute respiratory tract infections (ARTIs), of which only 52% were in accordance with guideline recommendations [13]. In this context, the CHANGE-3 study (Converting Habits of Antibiotic Use for Respiratory Tract Infections in German Primary Care, 2017-2020) applied a set of educative intervention components, of which several were delivered in digital formats: tablet computers in primary care practices, e-learning platforms for medical professionals, and a public website to promote awareness and health literacy among primary care physicians, their teams, and their patients (see Multimedia Appendix 1 for screenshots). A process evaluation carried out alongside the trial investigated the uptake and diffusion of these interventions, which were novel to the targeted physicians and practices. Figures 1 and 2 show examples of the topics covered on the study-specific website.

The diffusion of many innovations starts slowly and then accelerates before slowing again [14]. Diffusion of Innovations theory (DIT) describes the process through which innovations diffuse and become adopted through social networks in populations [14,15]. DIT can support research and program development in assessing and understanding processes that lead to the desired translation of new ideas and technologies into widespread practice [16]. This classic middle-range theory offers concepts and approaches that can explain receptivity of health care practices and policies by individuals and organizations [17]. At the same time, the diffusion approach may help connect “research-based innovations with their potential users in a knowledge-utilization process [14].”

To evaluate the uptake and acceptance of implemented digital information delivery solutions used in the CHANGE-3 study by physicians, care teams, and patients in primary care in Germany, this study integrated an approach based on DIT that addresses three major diffusion postulates: (1) specific characteristics of innovations influence their diffusion (Relative advantage, Complexity, Compatibility, Trialability, Observability), (2) communication channels and key individuals embedded in social networks play an important role, and (3) consequences of the uptake of innovations can be unanticipated. The study primarily focused on the digital information delivery components, as these comprise a new approach to promote awareness and health literacy in primary care in Germany with regard to converting habits of antibiotic use for ARTI.

### Objectives

The aim of this mixed-methods study was to assess the acceptance and uptake of digital interventions for education and information delivery to patients, physicians, and care teams with regard to converting habits of antibiotic use for noncomplicated ARTIs in primary care.

## Methods

### Study Design

This study is part of the process evaluation of the CHANGE-3 study, which was embedded in a two-armed cluster-randomized controlled trial with 57 practices randomized into each group (N=114). Randomization for the trial was stratified by rate of antibiotics prescriptions for ARTIs at baseline per practice and was performed by the Institute of Medical Biometrics and Informatics at the University Hospital Heidelberg [18]. Within the process evaluation and for this study, a mix of quantitative and qualitative methods was used in a convergent-parallel design [19-21] to gain insights into the working mechanisms of the intervention program, which aimed to convert habits of antibiotic use and strengthen health literacy competencies. Complementary interview guides and survey questionnaires were developed and used. Mixed-methods research combines qualitative and quantitative research elements with the aim of expanding and strengthening the conclusion and validity of a study [22]. By applying this design, a broad thematic spectrum could be analyzed to assess and understand factors relevant to the acceptance of digital information delivery with regard to converting habits of antibiotic use in primary care in Germany. The intervention components focused in this study comprised tablet devices with educational contents for patients, an e-learning platform for medical professionals which offered a communication skills training, and the study-specific website on rational usage of antibiotics for both groups (see Multimedia Appendix 1). The content provided via the tablet presented guideline-based educational videos and information and was adapted from the study-specific website [18,23] and from a similar application developed for a different study [24].

### Context

The comprehensive implementation program composed for the CHANGE-3 study (ISRCTN 15061174) was based on published research and experience in quality improvement programs [25,26], and it was tested in primary care settings. The overall aim of the CHANGE-3 study was to sustainably hinder the progress of antimicrobial resistance by promoting the conversion of antibiotic use habits and to increase health literacy in practice teams, patients, and the general public. To achieve this, a regional public campaign with multimedia approaches was used to encourage the general public’s engagement with rational antibiotic use and their active participation in a participatory decision-making process. In addition, an educational practice team intervention targeted internal process optimization in practices using educational intervention components, including digital information devices, individual feedback on prescribing habits, and outreach visits. The process evaluation conducted alongside the implementation program was embedded in the cluster-randomized trial, and we were particularly interested in documenting the uptake of interventions and understanding the mechanisms and impacts of the intervention components. The study was approved by the Ethics Committee of the University of Heidelberg (reference number S-349/2018). Participants in the process evaluation all gave written informed consent. Confidentiality and anonymity were ensured throughout the study. The planned outcome evaluation, which is not part of the process evaluation, will provide information about effects of the interventions on the prescribing of antibiotics for patients with respiratory symptoms. As the CHANGE-3 study only recently concluded, analyses referring to the outcome evaluation have not yet been completed.

### Survey

After the start of the intervention, all GPs (n=132) and a sample of nonphysician health professionals (n=208), comparable to medical assistants (MAs) in the United States [27], were invited to engage in a survey (T1) in May 2019. The participation of the MAs was restricted to a maximum of 2 per practice with the intention to limit imbalance in the sample and to ensure that the reimbursement budget would not be overdrawn. To be eligible for inclusion, the GPs and MAs were required to be participants in the intervention or control group in the CHANGE-3 study and to have mastered the German language. Based on constructs of the Theoretical Domains Framework (TDF) [28], a study-specific questionnaire was developed and used. It included tailored items to facilitate investigation and understanding of the mechanisms and impact of the intervention components as well as the contextual factors. No patients were invited to participate in this survey, as it focused on the practice teams’ perspectives on the applied intervention components. The questionnaire also included items referring to socio-demographic aspects and characteristics of the work environment. Email reminders were sent out after 4 weeks to increase the response rate. In March 2020, all participants who had returned T1 questionnaires were invited to participate in the follow-up survey (T2). No reminder was sent out.

### Interviews

Open-ended, semistructured, guide-based telephone interviews with the GPs and MAs participating in the CHANGE-3 study and a sample of their patients were conducted to explore their perspectives on the applied intervention components in general and the digital information delivery in particular. The interprofessional team of researchers (Health Services Research, Public Health, General Practice) developed study-specific interview guides for the three groups of interviewees (see Multimedia Appendices 2-4 for translated versions). Interview guides were based on constructs of the TDF [28], a literature review, and predefined research questions. All recruits were required to be at least 18 years of age, legally fully competent, and in fluent command of German. The potential recruits considered were GPs and MAs who were participating in the CHANGE-3 intervention group and were working in a primary care practice in the German federal state of Baden-Wuerttemberg or Mecklenburg–Western Pomerania. Invitations to participate in an interview were sent out via the aQua Institute, Goettingen, Germany. Patients who sought treatment for an ARTI during the intervention period at one of the intervention or control group practices were also eligible to participate in an interview. Using an opt-in approach, selected participating practices could support and initiate patient recruitment by addressing eligible patients. Support material was provided to care teams in these practices and facilitated a structured patient recruitment process.

All interested parties meeting the inclusion criteria received printed material as well as a telephone call from the research team at the Department of General Practice and Health Services Research, University Hospital Heidelberg, Germany, to provide further information. Participants were required to return a signed letter of intent to be included in the process evaluation and participate in the interview. In addition, the interviewees completed a one-time sociodemographic survey. All interviewees and care teams who supported patient recruitment received a small reimbursement fee. The first interview in each group served as a pilot. After that, minor adjustments were included where considered appropriate by the research team. No targeted sample size was set for the interviews, and data were collected until saturation of information was reached.

### Data Collection and Analysis

All T1 and T2 survey questionnaires returned to the Department of General Practice and Health Services Research at the University Hospital Heidelberg by June 2019 and April 20, 2020, respectively, were registered and pseudonymized by an experienced study nurse (T1) and the research team (T2). Support staff recorded all questionnaire data electronically in SPSS version 25 (IBM Corporation). Data were checked for plausibility by two study team members (RPD and LK), and typos were corrected where applicable. Subsequently, the data were analyzed descriptively in SPSS by the same two researchers. For this study, survey data referencing digital information delivery and sociodemographic characteristics were extracted from sets of items of both survey questionnaires (T1, T2) and included for analysis (see Multimedia Appendices 5 and 6 for the translated questionnaire items). Survey data were interpreted within the frame of applicable DIT postulates.

Applying a purposive strategy with regard to equal distribution of sex and region, all GPs and MAs in the intervention group were invited to participate in an interview. A sample of 39 interview participants was recruited by the CHANGE-3 study team at the Department of General Practice and Health Services Research, University Hospital Heidelberg, between November 2018 and April 2019. This strategy facilitated identification of individuals who were especially experienced with regard to the phenomenon of interest and supported a detailed understanding of key themes and relations to specific behaviors and roles [29]. All interviews were conducted via telephone by two members of the research team at the Department of General Practice and Health Services Research, University Hospital Heidelberg. Both researchers had profound experience in qualitative interviewing. All interviews were audio-recorded, pseudonymized, and transcribed verbatim. Data were managed with MAXQDA 2018.2 qualitative data management software (Verbi Software). The qualitative data were first thematically analyzed in a framework analysis based on constructs of the TDF and then combined with an inductive de novo approach according to concepts emerging from the data. To provide an explanatory model, and because most statements appeared to relate to social processes affecting diffusion, the identified key themes were subsequently categorized in accordance with three mirrored DIT postulates: (1) innovation characteristics, (2) communication channels, and (3) unanticipated consequences. All qualitative data generated from the interviews with the GPs, MAs, and patients, the field notes, and the sociodemographic survey were included for analysis. Quantitative and qualitative data were first analyzed separately and then brought together to complement each other.

## Results

### Principal Findings

Findings derived from both quantitative and qualitative data are presented with a focus on digital information provision via the intervention components “tablet,” “e-learning platform,” and “website” and are categorized in accordance with selected postulates derived from DIT [13,14]. The postulates relate not only to relevant contextual factors expected to be identified from the survey data but also to perceptions regarding the professional role, beliefs about the consequences of the innovation, and emotions, as derived from the qualitative data.

**Figure 3:**
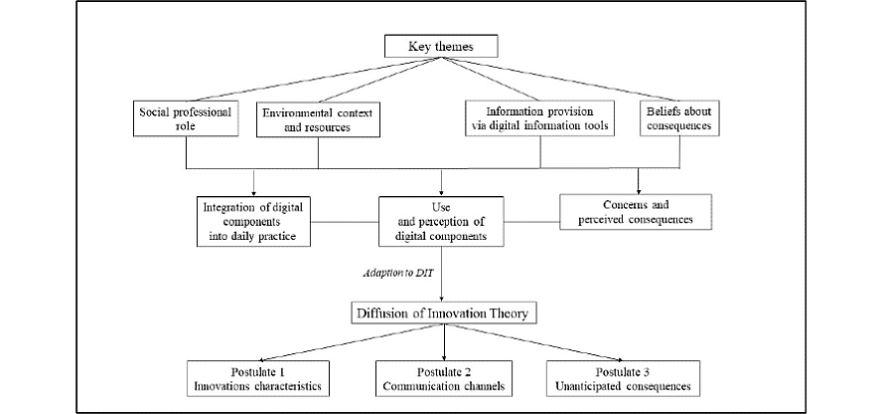
Theorizing analysis approach of the study. DIT: Diffusion of Innovations theory.

The theorizing analytical approach of the analysis is shown in Figure 3.

Key themes derived from the TDF provided the framework for the survey questionnaires and interview guides. Relevant contextual factors were identified from the survey data and qualitative data. During the qualitative analysis, the inductive domain “information provision via digital information tools” was added. All data were categorized according to the key themes and subsequently mirrored in the three DIT postulates where applicable to facilitate an explanatory framework for the findings.

### Sociodemographic Characteristics

A total of 80 GPs and 105 MAs returned the T1 questionnaire (N=185), and 63 GPs and 64 MAs returned the T2 questionnaire (N=127). In the intervention group, the mean age of the GPs was 53.2 years (SD 9.13) in T1 and 54.0 years in T2 (SD 9.70); in the control group, the mean age was 52.7 years (SD 9.6) in T1 and 56.5 years in T2 (SD 8.6). The mean age of the MAs was 40.9 years (SD 11.75) in both T1 and T2 in the intervention group; in the control group, the mean age was 42.0 years (SD 12.1) in T1 and 40.7 years in T2 (SD 11.4). The majority of the participating GPs in T1 were male (24/41, 598.5%, in the intervention group and 25/39, 64%, in the control group), and the MAs were almost exclusively female (50/50, 100%, in the intervention group and 54/55, 98% in the control group). For T1, 28/41 (68%) of the GPs in the intervention group and 34/39 (82%) in the control group were reported to have implemented changes in their practice routines in the last two years. Additionally, 15/41 (37%) of GPs in the intervention group and 19/39 (49%) in the control group stated that they had participated in previous antibiotics studies. Table 1 describes the characteristics of the participants in the intervention group and control group.

**Table 1:** Sociodemographic characteristics of the survey participants (T1) in this study (N=185).

Characteristic	GPs^a^ (n=80)		MAs^b^ (n=105)	
	Intervention group (n=41)	Control group (n=39)	Intervention group (n=50)	Control group (n=55)
Age, mean (SD)	53.2 (9.13)	52.7 (9.6)	40.9 (11.75)	42.0 (12.1)
Female sex, n (%)	17 (42.0)	14 (36.0)	50 (100.0)	54 (98.0)
Experience (years), mean (SD)	24.7 (9.6)	23.3 (9.2)	16.5 (11)	17.2 (11.2)
Implemented changes in the last 2 years, n (%)	28 (68)	34 (82)	34 (75)	38 (70)
Participated in another project to improve antibiotic prescribing, n (%)	15 (37)	19 (49)	14 (28)	32 (62)

^a^GPs: general practitioners.

^b^MAs: medical assistants.

Table 2 describes the sociodemographic characteristics of the interview participants (N=39). The mean age of the GPs was 53 years (SD 8.29), with a mean of 24 years of expertise (SD 8.2). Of the 16 interviewed physicians, 13 (81%) had a background in general practice, 2 (13%) had a background in internal medicine, and 1 (6%) was still undergoing specialty training. All the MAs were female (7/7, 100%). The mean age of the MAs was 48 years (SD 11.8), with a mean of 22 years of expertise (SD 11.8). The average years of employment in the GP practice in which they currently worked was 16.7 (SD 6.1). The mean size of the practice teams was 3 colleagues, GPs excluded. The mean age of the interviewed patients was 36 years (SD 12.2). On average, patients had been consulting their GP for eight years (SD 8). Of the 16 patients, 9 (56%) had ten years of school education.

**Table 2:** Sociodemographic characteristics of the interview participants in this study (N=39).

Characteristic	GPs^a^ (n=16)	MAs^b^ (n=7)	Patients (n=16)
Age, mean (SD)	53 (8.29)	48 (11.8)	36 (12.2)
Female sex, n (%)	9 (56)	7 (100)	10 (62.5)
Years of expertise, mean (SD)	24 (8.2)	22 (11.8)	N/A^c^
Years with current employer, mean (SD)	N/A	16.7 (6.1)	N/A
Years of consulting this GP, mean (SD)	N/A	N/A	8 (8)

^a^GPs: general practitioners.

^b^MAs: medical assistants.

^c^N/A: not applicable.

### Integration and Uptake of Digital Components Into Daily Practice

#### Survey Data

In the intervention group, T1 survey questionnaires were completed and returned by 41 GPs and 50 MAs. In the control group, 39 GPs and 55 MAs returned completed questionnaires. In total, 80/132 (60.6%) of GPs and 105/208 (50.5%) of MAs responded.

The uptake of the digital interventions was mixed and was overall limited. In the intervention group, 33/41 GPs (81%) and 36/50 MAs (72%) reported that they had not used the e-learning platform. Of the GPs who had used it, 7/41 (17%) felt motivated to prescribe antibiotics according to clinical guidelines. It was found that 9/41 GPs (22%) and 16/50 MAs (32%) found the tablet to be a helpful device in addressing patients’ expectations; meanwhile, 20/41 GPs (49%) and 19/50 MAs (38%) reported that they did not use the supplied tablets at all. When the tablets were offered to patients, 5/41 GPs (12%) and 13/50 MAs (26%) received the impression that patients actually used them and browsed the provided information. Of the 41 GPs, the study-specific website motivated 15 (37%) to engage in guideline-oriented antibiotic prescribing, helped 19 (46%) address patient expectations, and supported 16 (39%) in their therapy decisions. Some GPs also reported that using the website influenced therapy decisions (12/41, 29%) and led to a reduction in prescriptions (11/41, 27%).

The T2 survey questionnaires were returned by 32 GPs and 32 MAs in the intervention group and by 31 GPs and 32 MAs in the control group. In total, 63/80 (79%) of eligible GPs and 64/105 (61%) of eligible MAs responded. With regard to the e-learning platform, 10/32 GPs (31%) and 2/32 MAs (6%) reported using it. The website was visited by 14/32 GPs (44%) and 13/32 MAs (41%). Tablet devices were used by 15/32 GPs (47%) and 20/32 MAs (63%). Regarding the uptake of the devices, the T2 data confirm the T1 findings.

Table 3 provides information on the uptake of the three digital components as derived from the survey data. Table 4 and Table 5 provide information about the participants’ subjective assessment of the intervention components.

**Table 3:** Uptake of digital components in the intervention group of the study, n (%).

Digital device	T1 survey		T2 survey	
	GPs^a^ (n=41)	MAs^b^ (n=50)	GPs (n=32)	MAs (n=32)
Visited website	21 (51)	22 (44)	14 (44)	13 (41)
Used e-learning	8 (20)	14 (28)	10 (31)	2 (6)
Used tablet	21 (51)	31(62)	15 (47)	20 (63)

^a^GPs: general practitioners.

^b^MAs: medical assistants.

**Table 4:** Perceptions of the intervention component characteristics by the general practitioners, n (%).

Digital device	T1 survey (n=41)	T2 survey (n=32)
Website provides impulses for new behaviors, n (%)	N/A^a^	17 (56)
E-learning platform provides benefits in addressing patients’ expectations, n (%)	7 (17)	11 (34)
Tablet provides benefits in addressing patients’ expectations, n (%)	9 (22)	5 (16)

^a^N/A: not available.

**Table 5:** Perceptions of the intervention component characteristics by the medical assistants, n (%).

Digital device	T1 survey (n=50)	T2 survey (n=32)
Website is supportive for patient communication, n (%)	22 (44)	19 (59)
Website motivates me to support the GP^a^ more intensely, n (%)	17 (34)	19 (59)
E-learning platform motivates me to support the GP in treating ARTI^b^ infections, n (%)	11 (22)	6 (19)
Tablet is supportive of my daily routine, n (%)	18 (36)	16 (50)

^a^GP: general practitioner.

^b^ARTI: acute respiratory tract infection.

#### Interview Data

In the qualitative study conducted as part of the process evaluation of the CHANGE-3 study, a total of 39 telephone-based interviews were carried out with GPs (n=16), MAs (n=7) and patients (n=16). The average interview duration was 22.1 minutes (SD 6.21). Selected quotes supporting key statements are provided below with indications of the participant group and transcript position. Additional quotes are provided in Appendix 7.

### Uptake of Digital Components in Daily Practice

During the analysis of the interview data and the identified key themes, reluctance toward integrating the tablet intervention component into daily practice became apparent. In relation to DIT Postulate 1, “innovation characteristics,” the following section links this reluctance to use tablet devices with the five innovation characteristics from DIT.

The GPs raised concerns regarding the *compatibility* of their professional values with the digital devices. Of the 16 GPs interviewed, 6 (38%) articulated a conscious decision not to use the digital information tools. They saw their medical practice as a place of tranquility, where patients have the opportunity to calm down during times when they are constantly flooded with information. Moreover, 4/16 GPs (25%) raised additional concerns regarding the usage of the tablets, as they perceived themselves as the prior channel of information for patients and strongly saw themselves in the role of an adviser. They assumed that even younger patients would prefer advice from human beings, which may reflect an unclear *relative advantage* of the tablets. The MAs shared these opinions; however, they also addressed aspects referring to the quality of generally available information that is obtainable via digital sources. The patients suggested that using a television screen could be more appropriate for waiting areas. A subjectively perceived want of *observability* appeared to also foster reluctance toward using the devices. Referring to the required comprehension skills, one GP raised *complexity* concerns of potentially excluding older patients by using tablets; this GP mentioned that with the aim of equal treatment, tablets should not be used regularly for information delivery in primary care until a complete generation change has been accomplished. *Trialability* considerations did not seem to be relevant. In summary, the GPs expressed that the tablets did not play a major role in their approach to patient-centered information delivery.

I think people already are getting bombarded enough with this stuff. I don't want that in the practice here. We actually also have a ban on mobile phones here. So, of course you can play around on your mobile phone, but you must not talk on the phone [...] I believe that this is also quite good for the patients if they come to rest for a few minutes in the waiting area. In this respect, I don't see a place for it in my practice now.

GP 14, #64

And I also don't want my older patients in particular to have the impression that “This is something that I can't go along with anymore[...]”

GP 09, #21

In my opinion, we already have enough gadgets like computer, tablets and so forth where patients can inform themselves, and some of them show up here with preconceived opinions about their conditions and how to treat them. You have to be a little careful there.

MA 03, #46

I believe, it rather makes sense to offer a TV or something bigger where information is permanently running, because otherwise, just one person at a time is able to use it [tablet], which does not make a lot of sense then, or a constantly changing display of information everybody can follow would make more sense. A tablet doesn’t change much, I guess.

Patient 02, #32

### Use and Perception of Digital Intervention Components

Although the provision of the digital information tools was communicated to all participating GPs and MAs, their recollection of this was limited. The following section reflects statements from interviewed GPs and MAs who were aware of the presence of the digital information tools. We also include hypothetical statements from participants who did not encounter the intervention components. All these statements provide insight into the beliefs of the study participants about the appraisal of digital devices and the dissemination of information about these devices.

Awareness of the provided e-learning module was limited. Only 2 of the 16 interviewed GPs (13%) described in-depth use of the provided e-learning platform and considered it to be helpful in counselling situations. The communicative elements were seen to be helpful in dealing with patients’ potentially unwarranted expectations. Only one GP categorized the illustrated situations as unrealistic; however, they still considered them to be helpful to highlight specific situations. The MAs did not describe using the e-learning module at all.

Yes, very staged scene, but to make it clear to you, I thought it was good.

GP 06, #42

Ah yes, so you just say, let's say, not affront the patient somehow and so you just indirectly say that people who have certain expectations or who want to think an antibiotic now would just lead faster to their goal to get well again, so these communication-related aspects were very helpful.

GP 02, #18

The website was visited by only a few of the interviewees. Time constraints and a lack of awareness of the existence of the website were mentioned as the main reasons for not using it, pointing to potentially insufficient introduction and communication. However, the design of the website was described as visually appealing, yet overloaded with information. All the participants considered an educational website providing clear and relevant health-related information to be an asset; however, they assumed that age-related preferences were in place.

Yes, I am aware of it, but I did not visit it [website] yet.

MA 01, #78

No, somehow that [website] went right past me.

GP 18, #54

You could click on different topics, short and concise, attractively constructed, but I would have arranged it in a little less cluttered way, less information then. Yes, but overall it is attractively interesting, you readily want to engage with it. Well, “Oh, what’s this,” have a brief look.

GP 09, #9

Patients who had not yet had the opportunity to use the tablets were asked how they would envision provision of health-related information to patients in general practices. Of the 16 patients, 7 (44%) named tablet applications as potential information tools and demonstrated an open-minded attitude toward the digital provision of health-related information. Four of the 16 patients (25%) did not share their opinion, and 6 (38%) demonstrated a somewhat hesitant attitude toward digital devices. One patient saw the opportunity to use the tablet as a tool to prospectively retrieve information about the recommended usage of antibiotics. Regarding a potential lack of knowledge, this would provide the opportunity to avoid uncomfortable situations during consultation with the GP, potentially shorten the consultation, and allow more time to address unclear aspects and remaining questions. In contrast to this, another patient stated having no interest in health information provided by a tablet solution when having a cold and feeling sick. Furthermore, 5/16 patients (31%) considered televisions in waiting areas of medical practices to be a suitable alternative information tool. The website was considered to be an option for younger adults, and suggestions were made as to where such a site could be referenced.

I am not sure. I mean, when I’m sick, I am really sick and when I’m sitting there, I personally have no interest in reading information on tablets.

Patient 17, #77-78

By the help of information leaflet, right. It has to be printed on them so people can find the website. That’s a possibility that spontaneously popped into my head.

Patient 12, #73

In addition, another patient pointed out the difficulty of staying in line with the received information if a GP acts contrary to the recommendations. When in doubt about the treatment, standing up against a physician and refusing an antibiotics prescription was considered to be difficult, especially in situations where no laboratory results were available. In this scenario, to be on the reputed “safe side,” the patient would prefer to go along with the GP’s decision.

Because you go to the doctor and when he says: “Ah, well antibiotics are necessary,” then you say: “Yes, well, then I will take them.” So, I just have to come back to the ENT especially, because he hasn't taken a smear or anything else, for example, he just determined that: Chronic sinuses: antibiotics. And yes, it didn't do anything either.

Patient 03, #42

GPs and MAs noticed implications for their daily routines, which they attributed to participating in the study in general and to using the digital information devices in particular. Of the 7 MAs, 4 (57%) saw an impact of using the tablets on their daily routines. The main influence was seen in communication with patients. With the help of the devices, the MAs felt it was easier to convey the importance of antibiotics-free treatment to patients. Additionally, they saw the opportunity for patients to inform themselves prospectively, which was considered to lead to a decline in the number of necessary consultations. Additionally, the MAs described a broad range of assumptions and beliefs about the tablets’ influences on their own knowledge gain. This extended from the feeling of “no influence at all” to the feeling of having “increased confidence” regarding the usage of antibiotics. GPs saw changes in their communicative interactions with patients and considered their participation in the CHANGE-3 study to have led to notably more forthright debates about the relevance of antibiotics. They reported that they had started to address patients’ perceptions regarding the prescription of antibiotics more directly. Patients only hypothetically contemplated the potential effects of the digital provision of health-related information in primary care practices.

Well, you actually ask the patients more often, right? “Do you expect an antibiotic?”, you also say right away “viruses are not antibiotics accessible!”, you discuss that simply more offensively, right? You also assume somehow that it is clear, but to most people it is not clear at all, perhaps one is too much in one's own world and perception, right? - you simply integrate this more obviously in the conversation.

GP 01, #22

So, I think definitely better than before. I think you can always learn something and I definitely feel confident and literate [enough] in this topic to be able to also give the best possible advice to the patients.

MA 05, #34

### Concerns and Perceived Consequences

Unanticipated concerns and consequences which had not been considered beforehand emerged from the data and appeared to have a major impact on the participants’ reluctance to use digital information tools. To begin with, the influencing factors were organizational in nature and addressed daily routines in general practices; hygiene issues were mentioned as a major factor that obstructed the use of tablets. GPs as well as patients articulated concerns about using tablets in a public waiting area with regard to keeping them clean and disinfected. While patients’ concerns referred to the potential risk of infection, the GPs also contemplated the proper provision of disinfection wipes next to the devices. A noticeably low technology affinity led to reduced use of the tablets, the e-learning module, and the study-specific website. The e-learning platform provided video-based situations to all GPs in the intervention group to illustrate potential difficulties in provider-patient communication and in addressing patients’ expectations; however, it was only used by a small number of participants. The GPs and MAs did not extensively familiarize themselves with the study-specific website. Thus, it was referred to only on occasion, and patients were not systematically made aware of it.

Yes, I would ask myself: “How many people touched it before me?” I wouldn't touch that honestly. Well, and I don't know how often [...] it might get disinfected once a day, but if there are five people alone with a flu virus somehow and then it's not the same strain, yes, well [...] well, I do not support it.

Patient 11, #54

So, on the one hand I find it problematic, also because everyone is tapping on it and also from the hygienic aspect I should actually put disinfectant wipes next to it […]

GP 09, #21

Another unanticipated aspect was the perception of intensified time constraints in the usage of tablets. The MAs felt pressured to find time to incorporate the tablets into their daily routines. For them, the intervention component represented an additional task for which they felt responsible. The GPs and MAs both contemplated whether the amount of time a patient spends in the waiting area was sufficient for proper knowledge transfer. From their perspective, the value of tablets as an information tool did not justify investing the scarce resource of time into efforts to brief patients about the proper usage of the devices.

Well, that would tie up far too much time at the front desk, if a MA first has to explain what to do with the tablet, then she has to collect a deposit for it and then return it, disinfect it, that would tie up far too much MA working time, so we didn't use it.

GP 04, #22

[...] at our place, patients often don't have much time in the waiting area to deal with it [tablet] because they don't sit [and wait] that long […]

MA 07, #30

One further unexpected organizational aspect stemmed from a general concern of the GPs and MAs about the potential theft of the tablets. One MA recalled a moment when even a painting had been stolen from a wall in their practice. Under such circumstances, a tablet, as an object of higher value, was not considered to be the right information tool to distribute to patients in a medical practice. As a consequence, due to hygiene issues, a perception of intensified time constraints, and concerns about potential theft, the tablets were rarely used as intended or were not offered to patients at all.

Yes, and something has been stolen before, somehow a painting even from the wall, and we just didn't want to induce this stress with this tablet now, [...] we had this conversation in the practice […]

MA 01, #22

## Discussion

### Overview

The findings of this study in German primary care provide insights into the uptake of digital devices, medical professionals’ beliefs about digital educational interventions, and how these may have collided with the professionals’ understanding of their professional roles. The implementation met with challenges and hesitation, with the main barriers being perceived incompatibility with existing routines, low technology affinity, and concerns regarding the complexity of digital tools.

Active use of digital devices for health-related purposes appears to be more common in other high-income countries in Europe, such as Denmark and the Netherlands, where web-based platforms for patient education and e-learning for GPs have been established. Based on research evidence and prior experiences, the tablet application, the e-learning platform, and the website were implemented to strengthen health literacy, encourage guideline fidelity, and provide a subsequent change in antibiotic use habits for ARTI in German primary care. Prior studies used tablet computers to specifically disseminate health literacy in adults [30-32], for self-management programs for chronic diseases [33], diagnostics [34,35], interventions for parents and children [36-40], coping strategies after diverse surgeries [8,41], or as tracking functions for aims of weight reduction [42] and general fitness [43,44]. However, the findings of this study demonstrate a variety of challenges and a reluctance toward using digital information tools in German primary care practices for both patients and health professionals. This may be due to incompatibility of the digital devices with practice routines, patient expectations, and the perceptions of GPs and MAs about their medical practice and professional values. The GPs emphasized that they wanted to create a safe harbor for patients in a complex technological world, and they still saw themselves as the primary source of valid information for patients. As this attitude may stem from professional self-conception and may even be intensified by the age factor, future implementation programs should take these factors into account. MAs attempted to incorporate the digital information provision but considered it to be of little importance. Thus, not all study participants came into contact with the digital intervention components, which may also be explained by the choice of communication channels used in the public campaign and the practice team intervention for the distribution of the innovation. Patients considered the provision of health-related information via a trustworthy website to be a good option for younger patients and named options for creating awareness of such a website. They also saw waiting area televisions, which were not offered in the participating practices, as a better alternative to tablet devices. Adopters and nonadopters of the innovations voiced concerns about the applicability and adequacy of the digital components. In the next section, the findings will be discussed in relation to the selected DIT postulates to provide potential explanations for the findings.

### Postulate 1: Innovation Characteristics

Participants in this study found it challenging to implement the use of tablets into their organizational routines in primary care. The perceived lack of compatibility of this new intervention component with existing routines as well as the simple and convenient option to withdraw them appeared to be a major factor contributing to the absence of success. More importantly, a limited technology affinity and willingness to use digital solutions in the practice setting remained a major barrier. A qualitative study by Lyles et al [45] investigated tablets as a waiting room tool, where they were used as visit planners that focused on specific patient needs and were perceived as a “safe place” by patients to bring up sensitive topics. This supports our findings regarding the opportunity to avoid uncomfortable situations with GPs during consultations. Therefore, to disperse compatibility concerns, it may be beneficial to highlight the potential of tablet devices to maintain and support the desired “safe harbor” for patients.

Complexity concerns were also raised regarding the confrontation of older patients with modern technical devices. This is in line with the findings of a mixed-methods study by Patel et al [46] in which interviewed health care providers raised concerns about confronting older people with health literacy devices based on digital platforms. They surveyed 84 patients in a community health center waiting area in Massachusetts and identified a high level of interest in tablet-based health literacy solutions. Likewise, Stribling and Richardson [47] used tablets to provide educative health-related information in clinical waiting areas and surveyed patients afterward regarding their satisfaction with this method of digital information delivery. On average, the patients were satisfied with the usability and the educative input via the devices. However, in our study, complexity concerns for older people were primarily mentioned by health care providers or younger patients, not by the older people themselves. Future research should investigate perceptions of older patients more closely to evaluate if digital solutions are a reasonable intervention to strengthen their health literacy competencies.

The general possibility to observe the trialability effects of the new intervention [14] was not postulated sufficiently. It was assumed that the GPs would recognize the potential influence of the intervention on the health literacy competencies of patients. However, in light of known time constraints in primary care, it can be assumed that the GPs did not extensively evaluate the potential effects on patient health literacy and were not sufficiently aware of the inherent relative advantages. This leads to the assumption that there was no clear understanding of how the digital solutions could have been integrated into daily care routines and of the type of added value that could be expected. Instead, perceptions about innovation characteristics were more dominant, leading to a preference for more familiar and traditional solutions. Prior studies also used multimedia approaches to provide information in a digitally enhanced manner and offered printed information, videos, or individualized risk assessments [31,32]. In contrast to our findings, those studies showed significant effects of the use of tablets as educational devices for patients. It can be assumed that in the absence of significant impediments, broader adoption and use of the technology-mediated information delivery approaches could have been possible.

### Postulate 2: Communication Channels

The value of a new innovation must be defined and communicated. With Postulate 2, DIT suggests promoting new interventions with the help of personal approaches and the use of gatekeepers and opinion leaders. Rogers [14] stated that people make decisions based not only on rational considerations, but even more on their personal beliefs. Thus, in the primary care setting, it could have been more effective to demonstrate the value of the digital information tools using peer support instead of providing informative material that was based on rational facts alone. Also, peer patient coaching efforts could have been considered, as a lack of knowledge about antibiotic resistance may affect patients’ ability to understand the importance of the topic. Klingenberg et al [48] identified that less than 60% of patients in German primary care practices were aware of the possibility of being infected by resistant bacteria or the fact that antibiotics have no effects on viruses. To sufficiently transport knowledge and demonstrate the effectiveness and value of digital devices in achieving this transport, a more personal information delivery approach appears to be a relevant choice. However, with the exception of the offered outreach visits, such approaches remained largely unconsidered in the CHANGE-3 study, and gatekeepers were of no relevance.

### Postulate 3: Unanticipated Outcomes

The analysis of the qualitative data facilitated the identification of a number of unanticipated outcomes. Time constraints, insecurities, unclear procedures, hygienical aspects, and maintenance responsibilities were identified as barriers to sustainable application of the tablets in daily care. Reduced technology affinity greatly influenced the will and ability to use the digital information devices, the e-learning module, and the study-specific website. The e-learning platform was provided to all GPs in the intervention group. It consisted of video-based situations illustrating potential communicative difficulties between GPs and patients. The main focus was on addressing patients’ expectations, especially if they wished to receive antibiotic-based treatment without indication. Although this situation is often claimed to occur frequently, the e-learning module was only used by a small number of participants.

Schreiweis et al [49] conducted a systematic review in which they aimed to identify barriers to and facilitators of the implementation of e-health devices. The main barriers were found to be concerns about theft, absence of motivation, added workload, and general issues in adopting eHealth devices into organizational routines. Facilitators were found in the ease of use, staff motivation, involvement of all stakeholders, and the availability of resources. The findings in our study seem to match these results.

The reduction of antibiotic resistance is a goal that is preventive in nature. Noncompliant usage or prescription of antibiotics appears to have no directly perceived influence on individuals. Thus, this subjectively perceived lack of observability can lead to slower adoption of innovations. The effects of a desired behavior change become visibly lagged in time, which increases the difficulty of achieving satisfactory adoption of new intervention components with preventive characteristics. Therefore, the relative advantage of preventive innovations must be prioritized in communication [16]. Under such circumstances, it is even more important to point out the relevance of new and, here, digital intervention components, as awareness and responsibility help ensure a desired rate of implementation [50]. However, although the adoption of digital information devices was slow in this study, our findings show that the GPs and MAs noticed changes in communication and interaction with patients, and they attributed these changes to participation in the study and the use of its components.

Rogers [14] defined five adopter categories (innovators, early adopters, early majority, late majority, and laggards) that describe types of individuals and their willingness to adopt innovations. The criterion for building these categories was the degree of innovativeness at which an individual feels ready to implement an innovation into their professional routine. For sufficient contrast, a closer look at the characteristics of innovators and laggards is worthwhile. While innovators are defined by a more encouraged attitude toward change, laggards are driven by traditional values and suspicion. Laggards and late majority adopters require the removal of uncertainties about new ideas to decrease their skepticism and help them feel safe to adopt those ideas. According to the findings of this study, MAs and GPs who considered unanticipated consequences and compatibility concerns may have been driven by uncertainties or tradition and, thus, can potentially be categorized as laggards. According to Rogers, uncertainties should be addressed by individualized messages and involvement of opinion leaders. Because innovators are crucial to reach a critical mass and to accelerate the adoption of new ideas, it may also be a purposive approach to identify innovators and early adopters beforehand and let them act as role models in their social network of peers [14]. However, opinion leaders were not involved or identified in this study, and early adopters were not used to accelerate the adoption of the digital information devices.

This mixed-methods study aimed to assess the acceptance and impact of educative digital information delivery to patients, physicians, and care teams. The intention was to promote and facilitate a conversion of habits of antibiotic use for noncomplicated acute respiratory tract infections in German primary care. However, identified barriers impeded a broader use of digital solutions, thus pointing to a need to tailor and strengthen future implementation strategies in the field. This can minimize potential perceptions of burden on care teams and enable increased use, as potential barriers would be identified prior to implementation.

### Strengths and Limitations

The purposive sample of interview participants facilitated a detailed exploration and understanding of the central themes and underlying perceptions of the three participant groups. Structural variance in the qualitative data was ensured through age, gender, and years of working experience. Qualitative interviews are an important research tool to enable and foster understanding of perspectives of targeted groups. Telephone interviews can be seen as a valuable method of collecting information on sensitive topics [51]. All participants felt comfortable with the chosen method, as it accommodated their busy schedules. No participant opted for a face-to-face interview. Rapport was built without effort and was supported by frequent vocalized acknowledgments during the interviews. The participant-centered approach enabled consideration of verbal prompts, follow-up questions, and note-taking without distracting or influencing the interviewees. Applying this method resulted in rich data. Notably, DIT was developed before internet and digitalization efforts gained momentum in daily life and work, providing countless options for rapid dissemination of innovations. This may indicate a need to reassess the current applicability of DIT. However, applying DIT provided a useful explanatory frame for the hesitant integration of digital educational devices. Analysis of all data was guided by adequate methodological strategies aiming to minimize research bias and reduce the risk of losing relevant content. Typicality of the observations was shown by providing simple counts where their support of the findings can be expected. This also addresses potential issues of anecdotalism and exoticism. Reporting of the qualitative findings followed the recommendations of the COREQ (COnsolidated criteria for REporting Qualitative research) checklist [52].

Some limitations must be reported. Some members of the study population participated in a similar study that ran almost concurrently. This may have contributed to a stronger awareness of the topic but also to a clouded perception that impeded stronger engagement with the digital solutions, which were used by only a small number of participants. Social desirability of answers in the data cannot be excluded, although it can be considered to be less probable in light of the presented findings. Health literacy interventions have a preventive purpose, which may reduce fidelity rates. All presented findings must be interpreted with caution in terms of their representativeness.

### Conclusion

Patients and medical professionals in primary care can be reluctant to use digital devices for information and education that aim to strengthen the rational use of antibiotics in ARTIs. To encourage a higher rate of intervention fidelity, future studies should consider identified barriers and facilitators in a detailed manner, and more tailored interventions should be developed accordingly. Interventions that are in line with professionals’ perceptions will be crucial for success.

## Appendix

Multimedia Appendix 1 Screenshots of the tablet app, e-learning, and website.

Multimedia Appendix 2 Translated interview guide for GPs.

Multimedia Appendix 3 Translated interview guide for MAs.

Multimedia Appendix 4 Translated interview guide for patients.

Multimedia Appendix 5 Translated survey questionnaires for GPs.

Multimedia Appendix 6 Translated survey manuscripts for MAs.

Multimedia Appendix 7 Additional quotations.

## Abbreviations

ARTI: acute respiratory tract infection
CHANGE-3: Converting Habits of Antibiotic Use for Respiratory Tract Infections in German Primary Care Facilities
COREQ: COnsolidated criteria for REporting Qualitative research
DIT: Diffusion of Innovations theory
GP: general practitioner
MA: medical assistant
TDF: Theoretical Domains Framework
