# Supplementary material for: Applying Digital Information Delivery to Convert Habits of Antibiotic Use in Primary Care in Germany: Mixed-Methods Study
Source: J Med Internet Res. 2020 Oct 7;22(10):e18200. doi: 10.2196/18200 (PMC7578814; doi:10.2196/18200)
Supplement: Multimedia Appendix 1 [file jmir_v22i10e18200_app1.docx]

**Additional file 1**: Screenshots of the tablet application, e-learning and website


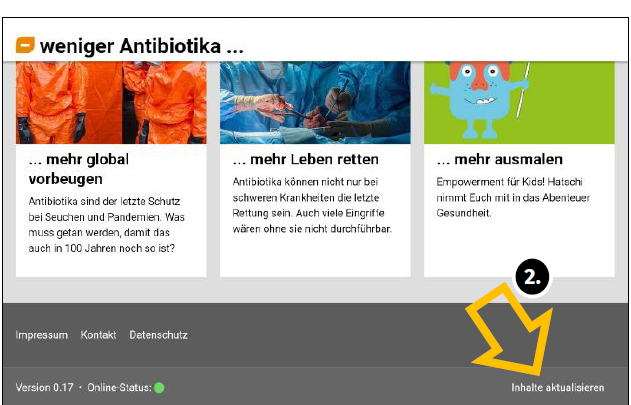


**Screenshot 1:** Tablet-App used in the CHANGE-3 study (German)
 less antibiotics …, … more global prevention, …saving more lives, … more coloring


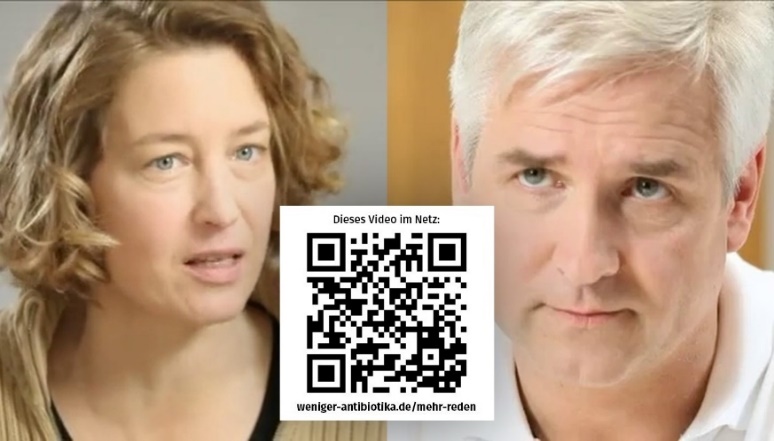


**Screenshot 6**: e-learning on provider-patient communication


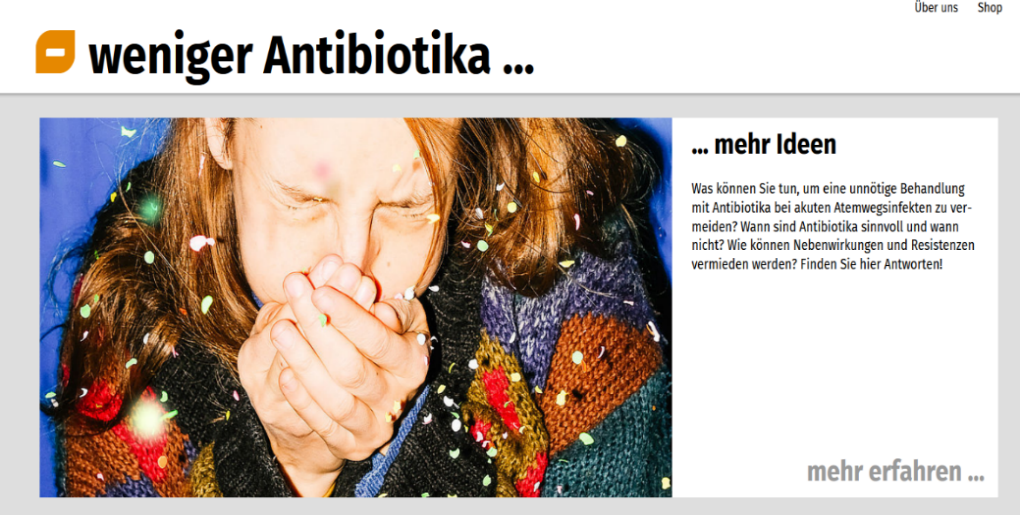


**Screenshot 2**: Study-specific website (German)
less antibiotics…, …more ideas


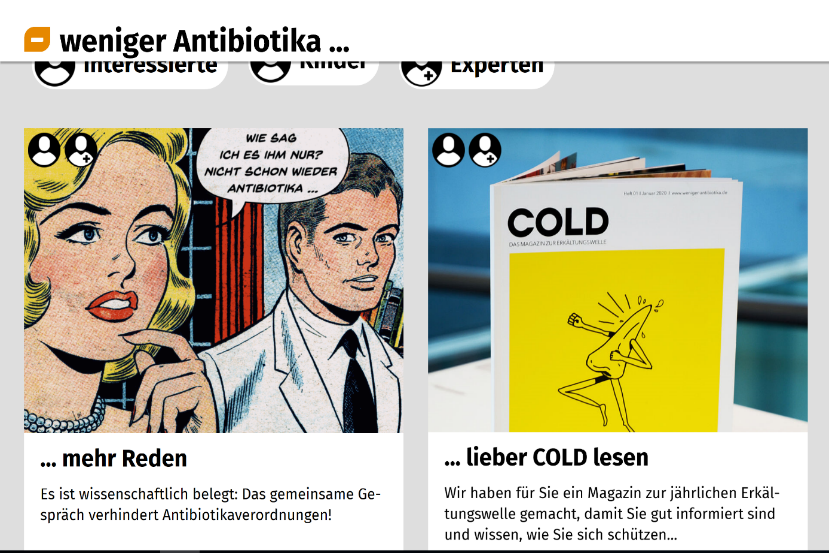


**Screenshot 3**: Study-specific website (German)
less antibiotics …, …more talk, … rather read the COLD magazine
