# Supplementary material for: Applying Digital Information Delivery to Convert Habits of Antibiotic Use in Primary Care in Germany: Mixed-Methods Study
Source: J Med Internet Res. 2020 Oct 7;22(10):e18200. doi: 10.2196/18200 (PMC7578814; doi:10.2196/18200)
Supplement: Multimedia Appendix 3 [file jmir_v22i10e18200_app3.docx]

**Additional file 3: Interview guide MAs (translated)**

**A – Exposure to antibiotics**

Please describe your range of tasks regarding patient care in your practice.

- Role of counselling

Which experiences did you make in dealing with patients with acute uncomplicated infections?

- Personal approach
- Communication with patients
- Strategies (recall system)

**B – Uptake and impact of offered intervention components**

Which of the intervention components do you consider helpful in treating patients with acute respiratory tract infections?

- Why / Explanation
- Adaption to personal work range
- Uptake of components / challenges regarding components

To what extent did the components support your daily work in the practice and in care of patients with acute respiratory tract infections?

- Change of range of tasks
- Change of patient interaction
- Change of communication in practice team

Personal perception of knowledge regarding antibiotics after implementing the intervention components?

- Updated knowledge / newly gained knowledge

**C – Dissemination of key messages**

Support by outreach visit and feedback report to bring campaign material closer to patients?

- Elements of public campaign considered important / less important
- Role of antibiotics resistance in daily work

Patient reactions observed?

- Patient expectations
- Organisational factors / communication in team / team structure

**D – Conclusion**

Which ideas and comments do you have for a further development of the interventions you got to know in the study?

- Recommendations for usage of antibiotics in patients with acute respiratory tract infections

What would you like to tell us besides already discussed topics?
