# Supplementary material for: Applying Digital Information Delivery to Convert Habits of Antibiotic Use in Primary Care in Germany: Mixed-Methods Study
Source: J Med Internet Res. 2020 Oct 7;22(10):e18200. doi: 10.2196/18200 (PMC7578814; doi:10.2196/18200)
Supplement: Multimedia Appendix 4 [file jmir_v22i10e18200_app4.docx]

**Additional file 4: Interview guide patients (translated)**

**A – Exposure to antibiotics**

Please describe what you typically do when you have a respiratory infection like a cough, a cold, a sore throat or hoarseness.

- Expectations of physician
- Role of antibiotics
- Influence of personal attitude on course of visit
- Experiences with household remedies

**B – Usage and impact of offered information material**

Which information material did you notice during your practice-visit?

- What was helpful? / Why?

How could you use the provided information and suggestions for yourself?

- Comprehensibility / relevance
- Resources needed to implement suggestions into daily life

During the visit you received information about your infection. To what extend might this information have an impact on your personal course of action with future respiratory tract infections like a cough, a cold, a sore throat or hoarseness?

- Transfer to other infections, e.g. urinary tract infection
- Change of attitude towards usage of antibiotics

How did you experience the communication with the practice-team and the care during your visit?

- Personal expectations about the visit

**C – Dissemination of key messages – public campaign**

Where did you notice the public campaign and its information about respiratory tract infections like coughs, colds or hoarseness and a considered usage of antibiotics?

- Positive aspects

Which messages of the campaign do you personally consider important and memorable?

- Changes of own behaviour

**D – Conclusion**

Ideas and comments regarding the topic of respiratory tract infections and antibiotics use?

What would you like to tell us besides already discussed topics?
