# Supplementary material for: Applying Digital Information Delivery to Convert Habits of Antibiotic Use in Primary Care in Germany: Mixed-Methods Study
Source: J Med Internet Res. 2020 Oct 7;22(10):e18200. doi: 10.2196/18200 (PMC7578814; doi:10.2196/18200)
Supplement: Multimedia Appendix 5 [file jmir_v22i10e18200_app5.docx]

**Additional file 5: Survey questionnaire T1 – GPs**

| **The study-specific homepage** [**www.weniger-antibiotika.de**](http://www.weniger-antibiotika.de) **(less-antibiotics)** | **Disagree strongly** | **Disagree** | **Neutral** | | **Agree** | **Agree strongly** |
| --- | --- | --- | --- | --- | --- | --- |
| … was visited by me |  |  |  | |  |  |
| … contains current information new to me |  |  |  | |  |  |
| … informs me in a comprehensible manner |  |  |  | |  |  |
| … strengthened me in dealing with patient expectations |  |  |  | |  |  |
| … I considered helpful for patient interaction |  |  |  | |  |  |
| … I recommended to my patients |  |  |  | |  |  |
| … strengthens my decision for or against prescribing antibiotics |  |  |  | |  |  |
| … has an impact on my interaction with patients |  |  |  | |  |  |
| … has an impact on the therapy patients with acute respiratory tract infections receive |  |  |  | |  |  |
| … lead to a decline in antibiotics prescriptions |  |  |  | |  |  |
| **The offered e-learning platform** | **Disagree strongly** | **Disagree** | | **Neutral** | **Agree** | **Agree strongly** |
| … was used by me |  |  | |  |  |  |
| … contains current information new to me |  |  | |  |  |  |
| … informs me in a comprehensible manner |  |  | |  |  |  |
| … strengthened me in dealing with patient expectations |  |  | |  |  |  |
| … I considered helpful for the patient interaction |  |  | |  |  |  |
| … motivates to follow a guideline-based treatment |  |  | |  |  |  |
| … strengthens my decision for or against the prescription of antibiotics |  |  | |  |  |  |
| … has an impact on my interaction with patients |  |  | |  |  |  |
| … has an impact on the therapy patients with acute respiratory tract infections receive |  |  | |  |  |  |
| … leaded to a decline in the prescription of antibiotics |  |  | |  |  |  |
| **The tablet with relevant information for patients** | **Disagree strongly** | **Disagree** | | **Neutral** | **Agree** | **Agree strongly** |
| … is available in the practice |  |  | |  |  |  |
| … is used by my patients |  |  | |  |  |  |
| … is helpful to discuss treatment options with patients |  |  | |  |  |  |
| … strengthened me in dealing with patient expectations |  |  | |  |  |  |
| … motivates to follow a guideline-based treatment |  |  | |  |  |  |
| … is relieving my decision for or against a antibiotics-therapy |  |  | |  |  |  |
| … gives me confidence in dealing with patients’ expectations |  |  | |  |  |  |
| … has an impact on my interaction with patients |  |  | |  |  |  |
| … has an impact on the therapy patients with acute respiratory tract infections receive |  |  | |  |  |  |
| … lead to a decline in the prescription of antibiotics |  |  | |  |  |  |

**Survey questionnaire T2-GPs**
