# Supplementary material for: Applying Digital Information Delivery to Convert Habits of Antibiotic Use in Primary Care in Germany: Mixed-Methods Study
Source: J Med Internet Res. 2020 Oct 7;22(10):e18200. doi: 10.2196/18200 (PMC7578814; doi:10.2196/18200)
Supplement: Multimedia Appendix 7 [file jmir_v22i10e18200_app7.docx]

**Additional file 7 – Quotes**

| Postulate 1: Innovation characteristics | “Even if young people like to use these [digital media], but when it comes to their body they just want to be noticed, too, want to be able to trust and that works in the end, otherwise they wouldn't even come here, then they could just use google, in the end they want to be looked at, touched and hear a human voice, that's why I'm not a friend of telemedicine or camera diagnostics, I think that has its very limits.” GP17#35  “But, in the end, it is talking to each other that makes the education work. And I have to say therefore most patients already know about it.” GP04#16  “In my opinion, we already have enough gadgets like computer, Tablets and so forth where patients can inform themselves. Beyond, some of them appear to have pre-consolidated expectations of what to do. You have to be a little careful there.” M03#46  “I believe, it rather makes sense to offer a TV or something bigger where information is permanently running, because otherwise, just one person at a time is able to use it [Tablet], which does not make a lot of sense. Several information sheets make more sense then, or a changing display of information everybody can read would make more sense. A tablet doesn’t change much, I guess.” P02#32 |
| --- | --- |
| Postulate 2: Communication channels | “Yes, very staged scene, but to make it clear to you, I thought it was good. So, I don't think anyone, I don't know whether someone is going to do this, this, this discussion situation so literally, so then and there, whether general practitioners are going to do it, well, whether this sentence, which kept coming back  "Other patients in this situation demand, well, or ask so" in such a way that you do it exactly like that, certainly not, well, but that you sometimes listen to what the patient actually expects, yes. That is a Yes.“ GP06#42  “No, I have to admit we did not explicitly refer to the Website” M05#42  “Oopps! I haven’t visited it [Website] yet.” GP10#29  “Yes, but too late. The study was almost over and thus, I did not use it [Tablet].” GP05#40  “I did not visit it [Website]. All of it is way too complicated for me. I do not like to work with those websites. I am just too old for it.” GP05#68-67  “I did not visit it. I believe, not many patients do it either. The worst thing is, it only makes sense when Dr. Google ranks the website on top of the list, right?” GP18#31  “Yes, we visited it once your colleague was here. Yes, I browsed it and considered it visually appealing but I did not work with it yet.” GP16#54  “Yes, I think there are too many websites and that’s kind of the problem […]. The question is how patients find access to the relevant information […]. I see it split in this information-flooding. It definitely might have a benefit but I can also imagine some of the patients are just swamped by it.” A16#58  „No, I did not notice it [Website]. It’s called less antibiotics, right?” P02#44  „Referring to my experiences, it perfectly fits for younger adults but not for the older ones because they are not used to the Internet.” P13#71  „I think it is okay for younger people, but not for older ones considering the fact that some of them do not have access to the Internet.” P12#64  „With the help of information leaflet, right. It has to be printed on it so people find the website. That’s a possibility that spontaneously popped into my head.” P12#73  “It became easier. You do not have to justify yourself anymore, right? There are always complainers, but of course this became less, right? All of this: “Why not? I guess you can do that!” It [the tablet] has improved that. MA01#50  “Well, with [the help of] these media, the patients probably already have answers to questions they would have asked us otherwise right? And probably don’t ask us for information that often anymore.” MA02#16  “So, I think nothing has changed, you've had your information before and I've monitored a little bit more than before, perhaps payed a little bit more attention to how many antibiotics were prescribed and I was surprised by how few people actually got an antibiotic.” MA02#20  “Yes, it's... you don't have to ask yourself anymore what [...]. You can read the information by yourself and still ask the doctor if you have any questions...ask if something is unclear.” Pat14#50-52  ‘Well, I thought it [the website] was attractively built by all means, I think [it was] easy to understand also for the patients, not highly scientific, but clear and explicitly phrased, I considered it to be positive.’ MA05#7  ‘… it [the website] perfectly fits for younger adults but not for the older ones because they are not used to the Internet.” Pat13#71 |
| Postulate 3: Unanticipated consequences | **“**I do not really like to lay out the tablet in the waiting area and to take care of it to be able to guarantee it works. I think it is nonsense; not necessary.” M07#28  “I personally think it is not necessary. The ones who want to inform themselves about these things in more detail can do it at home and use the internet. Because of short waiting-times most of our patients do not have enough time to deal with this.” M07#30 |
